# Essential m^6^A Methylation Regulator HNRNPC Serves as a Targetable Biomarker for Papillary Renal Cell Carcinoma

**DOI:** 10.1155/2022/9411692

**Published:** 2022-04-23

**Authors:** Jiajin Wu, Yuang Wei, Chenkui Miao, Songbo Wang, Xiaoyi Wang, Zengjun Wang

**Affiliations:** ^1^Department of Urology, The First Affiliated Hospital of Nanjing Medical University/Jiangsu Province Hospital, No. 300 Guangzhou Road, Nanjing 210029, China; ^2^Core Facility Center, The First Affiliated Hospital of Nanjing Medical University/Jiangsu Province Hospital, No. 300 Guangzhou Road, Nanjing 210029, China; ^3^Jiangsu Clinical Medical Research Institution, Nanjing 210029, China

## Abstract

m^6^A RNA modification is a common abundant posttranscriptional modification of mRNAs occurring in cancer growth and progression. Accumulated evidence has proved that HNRNPC, which acts as a m^6^A reader, plays an essential role in the promotion of cancer occurrence and development; nevertheless, the role of HNRNPC in papillary renal cell carcinoma remained to be discovered. In this study, we comprehensively identified HNRNPC as a hub gene involved in m^6^A modification in pRCC. Then, the expression level, survival outcomes, PPI network, function enrichment, immune cell infiltration, and single-cell analysis were performed. Finally, we found that HNRNPC significantly promoted renal cell carcinoma proliferation and migration in vitro. In conclusion, our work proved that HNRNPC may act as a momentous m^6^A regulator, as well as a potential targetable biomarker for pRCC.

## 1. Introduction

Renal cell carcinoma (RCC) is one of the most common malignant tumors in genitourinary system worldwide, which raised 79000 estimated new cases in the United States according to cancer statistic 2022 [1]. RCC encompasses three major histological subtypes: clear cell RCC (ccRCC), papillary RCC (pRCC), and chromophobe RCC (chRCC). Among them, pRCC is the most common nonclear cell RCC (nccRCC), accounting for 10-15% of all RCCs, and is associated with poor prognosis [2, 3]. Papillary renal cell carcinoma (pRCC) can be classified into two subtypes on the basis of genetic background: type 1 pRCC is characterized by MET alterations, and type 2 pRCC features alterations in CDKN2A, SETD2, BAP1, PBRM1, FH, NF2, TFE3, NFE2L2, and ARE [4–6]. Oncogenic mutation of MET is rare but identified as an essential characteristic of the pathogenesis of pRCC forms [6, 7]. However, detailed background of its derivation and progression has not been intensively studied. Therefore, collaborative studies become a pressing demand for discovering the deeper molecular mechanisms of pRCC, and the establishment of predictive biomarkers is urgently needed to guide clinical treatment and drug development.

N6-Methyladenosine (m^6^A) RNA modification is one of the most abundant posttranscriptional modifications of mRNAs occurring in cancer growth and progression [8–10]. mRNAs can be dynamically methylated and demethylated by specific methyltransferases (m^6^A writers) and demethylases (m^6^A erasers). Additionally, m^6^A is deposited in native RNA transcripts and posttranscriptionally mediate recruitment of downstream functional complexes through altering RNA structure or specific recognition by m^6^A-binding proteins (m^6^A readers), respectively [10, 11]. Accumulating evidence has confirmed the influence of the participation of m^6^A modifications in tumor proliferation and metastasis [12–14]. Chen et al. reported that m^6^A could impact the tumor microenvironment and prognosis of pRCC [15]. Despite the increasing application of m^6^A RNA modification in the field of cancer research in recent years, the studies on underlying mechanisms of m6A modifications in tumor development, especially in pRCC, are lacking.

As a member of heterogeneous nuclear ribonucleoproteins (hnRNPs) family, heterogeneous nuclear ribonucleoprotein C (HNRNPC) is an RNA-binding protein that assembles onto newly created transcripts in the nucleus of a eukaryotic cell [16]. Two distinct splice isoforms have been identified as hnRNPC1 and hnRNPC2, differing from each other by 13 amino acids [16]. Unlike other m^6^A regulators, after recognizing m^6^A modification, HNRNPC could selectively regulate mRNA abundance and splicing to transform the secondary structure of RNA [14, 17–19]. A great number of research reported that HNRNPC plays an essential role in the promotion of cancer occurrence and development. Take breast cancer, for example, HNRNPC is required for BRCA gene expression and homologous recombination [20]. Besides, HNRNPC regulates cancer-specific alternative cleavage and polyadenylation profiles in colon cancer [21]. However, the function of HNRNPC in pRCC still remains to be adequately discovered.

In the present study, we first investigated HNRNPC as a hub gene involved in the m^6^A modification in pRCC. Then, the expression level and survival outcomes were analyzed together with clinical characteristics as well as somatic mutation. PPI network, function enrichment, and correlation analysis with immune cell infiltration were performed to explore potential molecular mechanisms. Finally, we found that HNRNPC significantly promoted renal cell carcinoma proliferation and migration in vitro. In conclusion, our work proved that HNRNPC may act as a momentous m^6^A regulator, as well as a potential biomarker for pRCC.

## 2. Materials and Methods

### 2.1. Data Source and Preprocessing

Normalized RNA-seq data (FPKM format), somatic mutation, SCNA profiles, and corresponding clinical annotations of patients with papillary renal cell carcinoma from The Cancer Genome Atlas (TCGA, https://portal.gdc.cancer.gov), GTEx, and Gene-Expression Omnibus (GEO) database GSE15641 were integrated and analyzed. GSE15641 includes 23 normal kidney samples and 11 papillary renal cell carcinoma samples [22]. Somatic mutation, SCNAs, and clinical information including age, gender, pathological stage, TNM stage, primary therapy outcome, and survival information were included in this research.

### 2.2. Kaplan–Meier Survival Analysis and ROC Curve of HNRNPC in pRCC

Based on the median expression of HNRNPC, pRCC patients were divided into high HNRNPC expression group and low HNRNPC expression group. By Kaplan–Meier (K–M) method and the log-rank test, we evaluated the disease-specific survival (DSS), overall survival (OS), and progress-free interval (PFI) differences between these two groups. The receiver-operating characteristic curves (ROCs) were performed by using the R “survivalROC” package, and the area under the curve (AUC) values were calculated to assess the specificity and sensitivity. The nomogram was designed to predict survival of pRCC patients using R “rms” and “survival” packages.

### 2.3. Visualization of PPI Network

Protein-protein interaction (PPI) network was constructed using the STRING online database (http://string-db.org) and ComPPI database [23, 24]. Cytoscape software and Metascape website (https://metascape.org) were further applied to visualize and achieve gene enrichment analysis and coexpression network analysis [24]. Here, we used the R package “limma” to search the positive and negative 20 coexpression genes of HNRNPC based on the data from TCGA-KIRP.

### 2.4. Univariate and Multivariate Cox Hazard Regression Analyses

In order to identify independent prognostic factors, univariate and multivariate Cox regression analyses were utilized to exclude clinical characteristics with little prognostic values with OS, DSS, and PFI from age, grade, stage, and HNRNPC mRNA expression level.

### 2.5. Gene Set Enrichment Analysis (GSEA)

GSEA was performed to analyze the pathways enrichment between two groups divided by the median expression value of HNRNPC. The genesets were downloaded from the Molecular Signatures Database (MSigDB http://software.broadinstitute.org/gsea/msigdb/) [21]. Immunotherapeutic signatures, including oncogenic pathways which target therapy-associated gene signatures and predict radiotherapy responses, were also integrated [25]. The ssGSEA enrichment scores of these signatures were calculated using the “GSVA” R package [26, 27].

### 2.6. Single-Cell RNA Sequence Processing

Firstly, raw data of GSE152938 was downloaded from GEO database, and R package “Seurat” was used to process data (https://satijalab.org/seurat/) [28–30]. Dataset GSE152938 contains one pRCC sample and 11821 cells [28]. By filtrating with the criteria of >20% mitochondria-related genes or less than 1000 genes expressed or greater than 20,000 genes expressed, a total proportion of 9941 cells were finally obtained for further analysis. After quality control, we normalized the data and rescaled all the RNAs. Next, respective reduction of cell clustering, including tSNE and PCA, was performed, and cell cluster was obtained through the tSNE method. Finally, we used the “SingleR” package to get the cell type for cell population annotation [31].

### 2.7. Cell Culture and Quantitative Real-Time PCR (qRT-PCR)

The renal cancer cell lines (786-O, 769-P, ACHN, Caki-1, and Caki-2) and the human renal tubular epithelial immortalized cell line (HK-2) were purchased from the Type Culture Collection of the Chinese Academy of Sciences (Shanghai, China) and cultured in RPMI 1640 (786-O, 769-P), DMEM (ACHN), McCoy's 5A (Caki-1, Caki-2), and DMEM/F12 (HK-2) (Gibco, Thermo Fisher Scientific, USA) containing 10% fetal bovine serum (FBS; Gibco, Thermo Fisher Scientific, USA) and 1% penicillin/streptomycin (Gibco, Thermo Fisher Scientific, USA). All cells were cultured at 37°C in a humidified incubator containing 5% CO_2_. 293-T cells were transfected with control siRNA and siRNA-HNRNPC using Lipofectamine 3000 (Invitrogen, Thermo Fisher Scientific, USA). The lentiviral vectors containing overexpression of HNRNPC were cotransfected with pMD2.G and psPAX2 packaging vectors. Lentiviral transduction was performed in 769-P and Caki-2 cell lines. After selection with puromycin, stable overexpression HNRNPC cells were established.

The total RNA was extracted using Invitrogen TRIzol reagent (Thermo Fisher Scientific, USA) and subsequently reverse transcribed into cDNA using HiScript III All-in-one RT SuperMix (Vazyme, China). qRT-PCR was performed with SYBR qPCR Master Mix (Vazyme, China) using StepOne Plus (Applied Biosystems, USA) and LightCycler 480 PCR instrument (Roche Diagnostics, Switzerland) according to the manufacturer's instructions. The primers and siRNA Oligo used were listed in Table S1.

### 2.8. Clinical Samples and Immunohistochemistry (IHC)

pRCC samples and adjacent normal renal samples were obtained by radical nephrectomy. All diagnoses were confirmed by two independent senior pathologists. Informed consents of all patients were acquired in the study. The study design and protocol were approved by the ethic committee of hospital. IHC staining was performed as previously described [32, 33]. Briefly, the primary antibody was incubated as follows: anti-HNRNPC (Proteintech, 1: 200, China). The results of IHC staining with HNRNPC were detected and captured using a microscope (Olympus, USA).

### 2.9. Cell Proliferation and Colony Formation Assays

Pretreated cells were cultured into a 96-well plate at a density of 1.5 × 10^3^ cells/well. Cell proliferation was measured after 24 h, 48 h, 72 h, and 96 h using the CCK-8 Cell Counting Kit (Vazyme, China). The absorbance was measured at 450 nm with a microplate reader following incubation at 37°C for 1 h according to the manufacturer's protocols.

For the colony-formation assay, pretreated cells were seeded into 6-well plates (1000 cells/well). The cells were incubated for 10 days. Colonies were fixed in 4% paraformaldehyde for 20 min, washed with PBS twice, and stained with 0.1% crystal violet for further analysis.

### 2.10. Transwell Cell Migration Assay

A total of 1.5 × 10^4^ cells pretreated cells were seeded into the 24-well transwell upper chambers with serum-free medium for the migration assays. Medium containing 20%FBS was added to the bottom chamber. After incubation at 37°C for 36 h, the cells were fixed in 4% paraformaldehyde for 20 min and stained with 0.1% crystal violet. Cells were captured on a microscope in five randomly selected fields, and all of the experiments were repeated three times.

### 2.11. Statistical Analysis

All statistical data and figures were analyzed using R software (v4.1.3; https://www.r-project.org/) and GraphPad Prism 9.0 (San Diego, USA). The correlations between different genes were analyzed by Pearson's or Spearman's correlation analysis. The association between clinicopathologic information and HNRNPC mRNA expression was estimated by the Wilcoxon signed-rank test and logistic regression. All statistical results with *p* < 0.05 were considered statistically significant.

## 3. Results

### 3.1. Landscape of m^6^A RNA Methylation Regulators in pRCC

**Figure 1:**
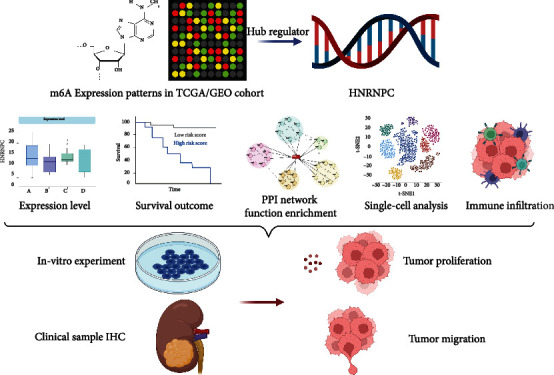
The flowchart of this present study.

**Figure 2:**
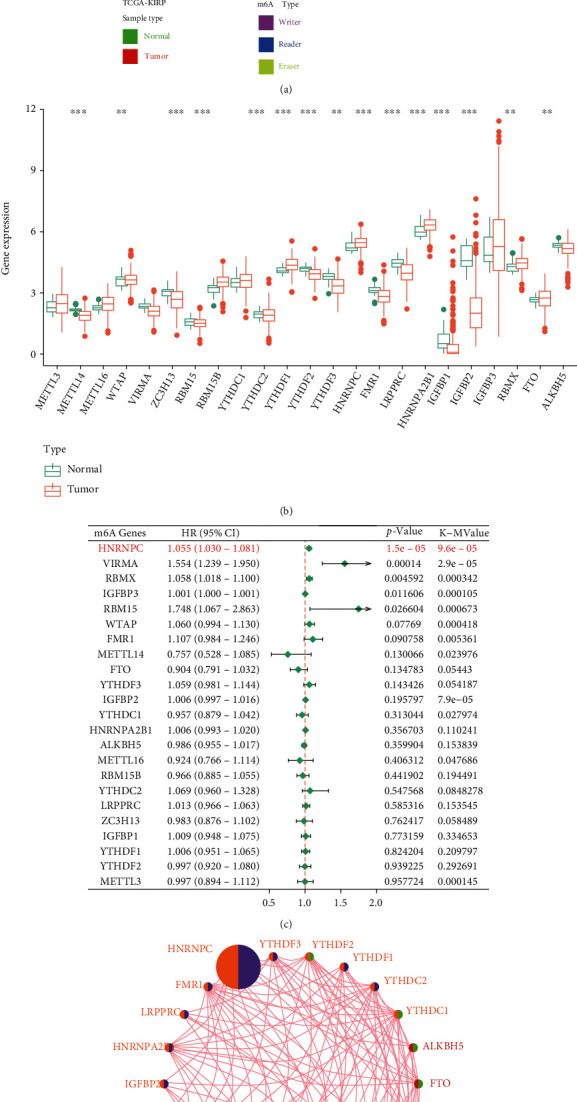
Landscape of m^6^A RNA methylation regulators in pRCC. (a) A heatmap exhibited the expression characteristics and of different m^6^A subtypes. (b) Boxplot showed the expression level of these m^6^A RNA methylation regulators. (c) Univariate Cox regression and Kaplan–Meier survival analysis. (d) Correlation network illustrated the interactions among m^6^A regulators. ^∗^*p* < 0.05; ^∗∗^*p* < 0.01; ^∗∗∗^*p* < 0.001; ns: no significant.

The flowchart of this present study is illustrated in Figure 1. Based on published studies, 23 common m^6^A RNA methylation regulators were filtered out, including 8 writers, 13 readers, and 2 erasers. The heatmap showed the expression characteristics and different m^6^A subtypes of these regulators in pRCC tissues compared to normal kidney tissues (Figure 2(a)). As shown in Figure 2(b), in total, 16 m^6^A regulators were significantly differentially expressed. Besides, univariate Cox regression and Kaplan–Meier survival analyses revealed that HNRNPC, VIRMA, RBMX, IGFBP3, and RBM15 were independent predictive genes, whereas other regulators were not associated with the prognosis of pRCC patients (Figure 2(c)). Ultimately, to further explore the natural interactions among m^6^A regulators, the correlations between 23 m^6^A regulators were investigated. The m^6^A network depicted a comprehensive landscape of m^6^A regulator interactions, correlation lineages, and their prognostic value in pRCC patients (Figure 2(d)). It is worth mentioning that HNRNPC was the most significant hub gene and risk factor. Accordingly, above results confirmed that HNRNPC was an upregulated m^6^A RNA methylation regulator as a hub gene and could predominantly predict the prognosis of pRCC patients. Hence, HNRNPC was chosen as the hub gene for further research.

### 3.2. HNRNPC Expression Was Elevated and Associated with Poor Clinical Outcome in Papillary Renal Cell Carcinoma

**Figure 3:**
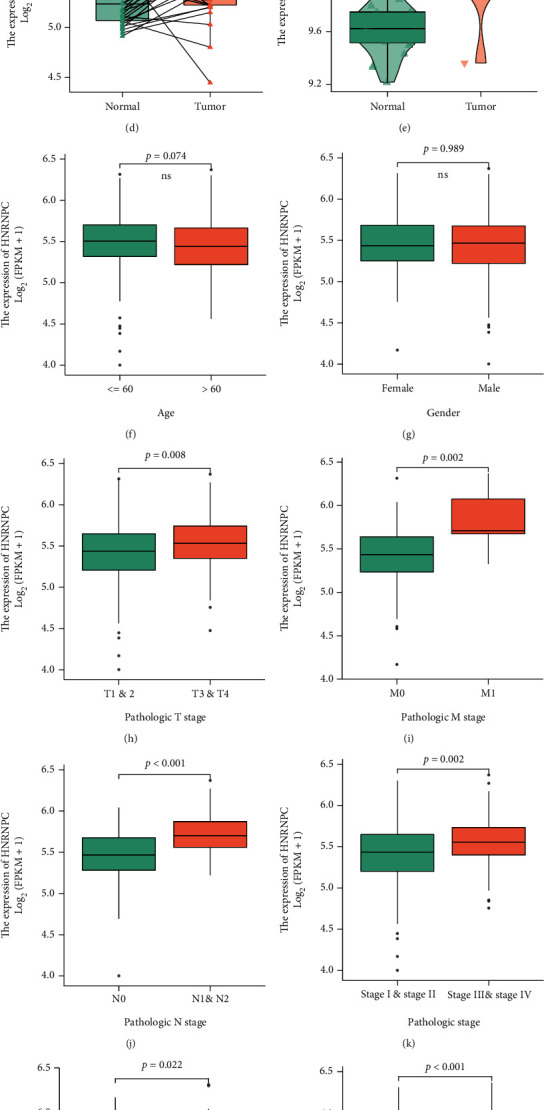
The expression level of HNRNPC in pRCC tissues and clinical characteristics. (a) The expression level of the HNRNPC in distinct tumors or specific tumor subtypes. (b) The expression level of HNRNPC in TCGA-KIRP and GTEx database. (c) The expression level of HNRNPC in TCGA-KIRP cohort. (d) The expression level of HNRNPC in TCGA-KIRP paired samples. (e) The expression level of HNRNPC in GSE15641 cohort. (f–o) The expression level of HNRNPC was analyzed by different clinicopathologic characteristics. (f) Age. (g) Gender. (h) Pathologic T stage. (i) Pathologic M stage. (j) Pathologic N stage. (k) Pathologic stage. (l) Primary therapy outcome. (m) OS event. (m) DSS event. (o) PFI event. ^∗^*p* < 0.05; ^∗∗^*p* < 0.01; ^∗∗∗^*p* < 0.001; ns: no significant.

To explore the role of HNRNPC in tumorigenesis, TCGA and GTEx databases were investigated to determine the expressing patterns of HNRNPC in different tumors. The results showed that HNRNPC expressed anomalously in various tumor tissues (Figure 3(a)). Validation of above findings in TCGA and GTEx databases indicated that the HNRNPC mRNA level was upregulated in pRCC tissue compared with normal kidney tissues (*p* < 0.001, Figure 3(b)). Meanwhile, samples from TCGA database alone also revealed high expression of HNRNPC in pRCC (*p* = 0.005 for unpaired samples and *p* = 0.028 for paired samples, Figures 3(c) and 3(d)), which was consistent with the external validation in independent dataset GSE15641 from GEO database (Figure 3(e)). To find the relationship between HNRNPC expression and clinical features, complete clinical and survival information of 389 pRCC patients was collected from TCGA database. After grouping patients by clinicopathological features, it could be observed that HNRNPC expression exhibited a significant increase in diverse subgroups of TNM classification and pathologic stage (Figures 3(f)–3(k)). Besides, HNRNPC was significantly upregulated in progressive disease and stable disease (PD&SD) and dead patients, compared to partially response and complete response (PR&CR) and surviving patients, respectively (Figures 3(l)–3(o)). In the subsequent analyses, median cut was used to dichotomize 389 pRCC individuals into high-HNRNPC (*n* = 145) and low-HNRNPC (*n* = 144) subgroup based on mRNA expression level. As shown in Table 1, HNRNPC expression was significantly correlated with the T stage (*p* = 0.043), N stage (*p* < 0.001), and M stage (*p* = 0.010). Furthermore, logistic regression analysis was adopted to describe the exact correlativity between HNRNPC expression and clinicopathological characteristics (Table 2). Taken together, above results suggested that HNRNPC may play an important role in promoting the malignant phenotype of pRCC.

### 3.3. HNRNPC Displayed Excellent Predictive Value in the Diagnosis and Prognosis of Papillary Renal Cell Carcinoma

**Figure 4:**
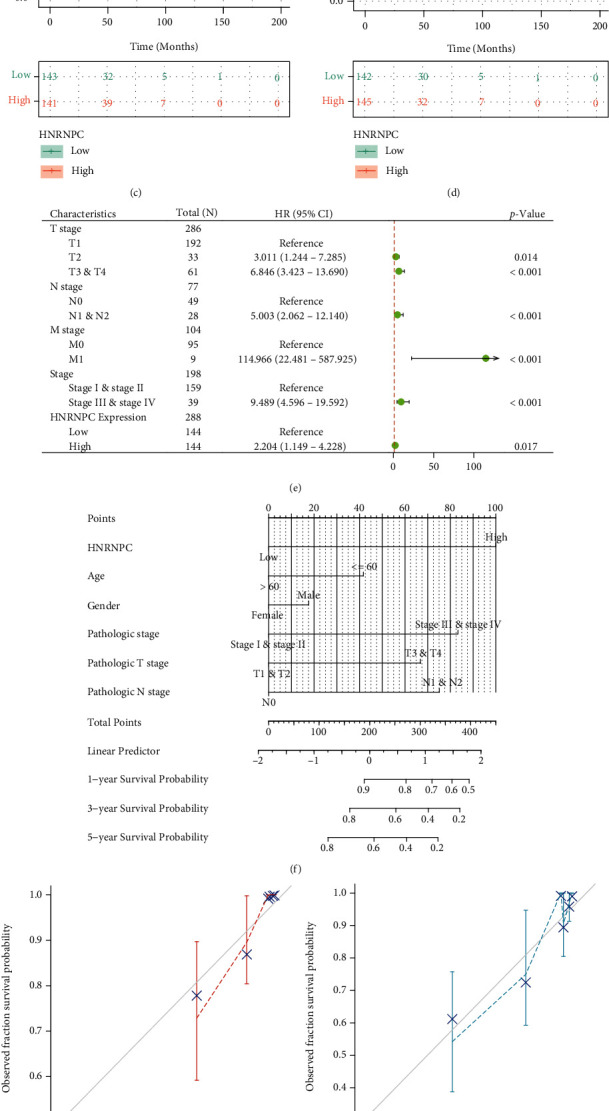
Survival analysis of HNRNPC and construction of nomogram. (a) ROC curve showed the efficiency of HNRNPC mRNA expression to distinguishing pRCC tissue from normal tissue. (b–d) Kaplan–Meier curve showed the prognostic value of HNRNPC in OS, DSS, and PFI. (e) Univariate Cox regression analysis in overall survival (OS). (f) Construction of a nomogram for estimation of survival rates for pRCC patients. (g–i) Calibration curves displayed considerable concordance between observed survival probability and the predicted results for 1-, 3-, and 5-year.

To explore the predictive value of HNRNPC, ROC curve was drawn to demonstrate its capability of assisting the diagnosis of pRCC. As shown in picture, the area under the curve (AUC) of ROC was 0.650 (95% CI 0.554–0.747; Figure 4(a)), indicating that HNRNPC possessed respectable sensitivity and specificity for the diagnosis of pRCC patients. Next, Kaplan–Meier survival analysis was applied to verify the predictive value of HNRNPC on clinical outcomes. As shown in Figures 4(b)–4(d), overall survival (hazard ratio (HR) = 2.46, *p* = 0.008), disease-specific survival (HR = 3.18, *p* = 0.008), and progression-free interval (HR: 2.31, *p* = 0.003) in high-HNRNPC groups were all statistically worse than those in the low-HNRNPC group. Moreover, a univariate Cox regression analysis was performed to further evaluate the predictive value of HNRNPC on overall survival (OS). In addition to the clinical stage, T stage, N stage, and M stage, HNRNPC expression turned out to be an important independent risk factor for OS as well (HR: 2.204, 95%CI = 1.149‐4.228, and *p* = 0.017, Figure 4(e) and Table S2). Ultimately, to provide a more accurate estimation of survival rates for pRCC patients, six factors were incorporated into a prognosis model as shown in the nomogram (Figure 4(f)). Besides, calibration curves displayed considerable concordance between observed survival probability and the predicted results for 1-, 3-, and 5-year. These results were largely consistent with the actual 1-, 3-, and 5-year survival in the TCGA-KIRP cohort (Figures 4(g)–4(i)).

### 3.4. Genetic Mutation Frequency of HNRNPC

Considering the pathogenic nature of gene mutations, the cBioPortal database was investigated to obtain the mutation frequencies and status of genetic alterations of HNRNPC across multiple cancer types. It was found that amplification was the main frequent genetic alteration followed by deep deletion in TCGA and MSK-IMPACT cohort [34]. However, unlike other carcinomas, deep deletion and mutation were the two most common subtypes in pRCC patients, which contributed an alteration frequency of ∼2% (Figure S1A-B). The mutation frequency sites and case numbers of the HNRNPC genetic alterations were detected locating between amino acids and displayed in Figure S1C. Furthermore, using TIMER database, the copy number variation (CNV) of HNRNPC, including deep deletion, arm-level deletion, diploid/normal, and arm-level gain, significantly affected the infiltration levels of B cells, CD4+ T cells, CD8+ T cells, neutrophils, macrophages, and dendritic cells in pRCC [35] (Figure S1D).

### 3.5. PPI Network, GSVA, and Functional Enrichment Analysis of HNRNPC

**Figure 5:**
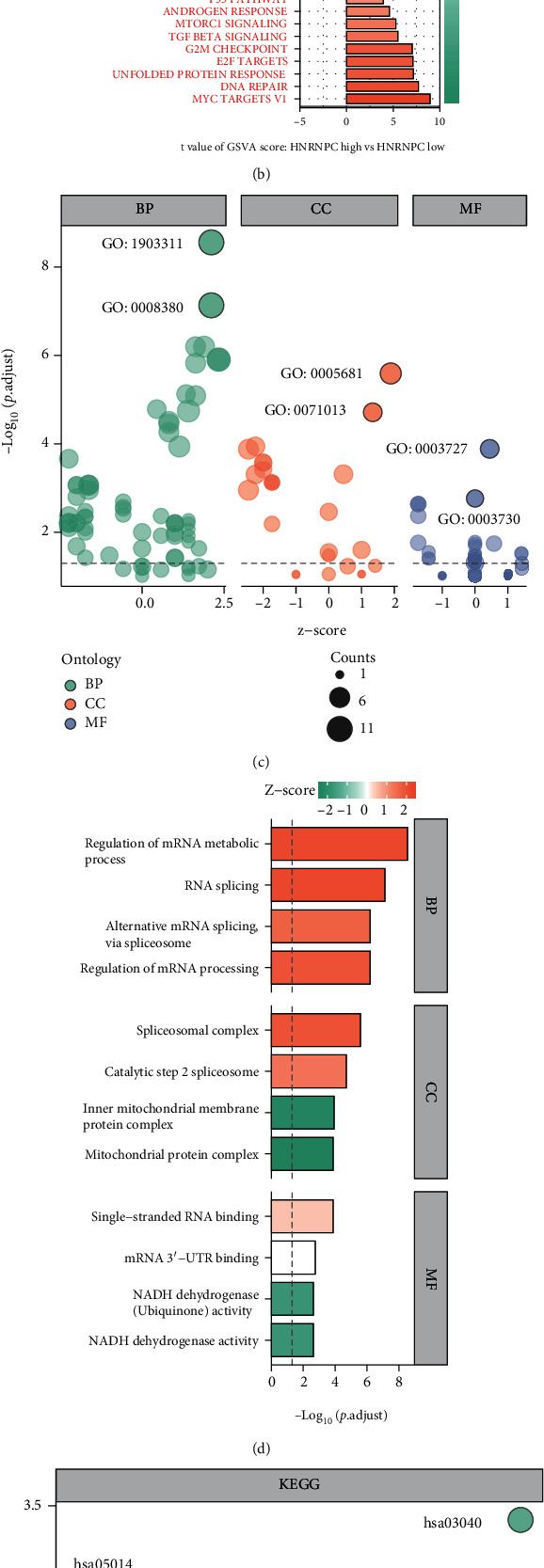
PPI network and functional enrichment analysis. (a) ComPPI database was investigated to obtain a cellular compartment-specific protein-protein interaction network of HNRNPC. (b) GSVA illustrated that HNRNPC participated in several Hallmark pathways. (c, d). GO analysis of HNRNPC-related genes. (e, f) KEGG pathways enrichment of HNRNPC-related genes.

To investigate the underlying molecular mechanism through which HNRNPC elicits tumorigenesis in pRCC patients, further analyses were conducted on protein interactions and gene functions. STRING and GeneMANIA databases were used to help establishing the PPI network and further explore the interaction of HNRNPC-related genes [36] (Figure S2). Meantime, ComPPI database was investigated to obtain a cellular compartment-specific protein-protein interaction network as well [23] (Figure 5(a)). Next, GSVA was employed to identify pathway differentiations among paired samples derived from TCGA-KIRP cohort. The results illustrated that HNRNPC participated in several Hallmark pathways including “MYC targets,” “DNA repair,” “E2F targets,” and “G2M checkpoint” (Figure 5(b)). After selecting top 15 HNRNPC coexpressed genes, the function enrichment and pathway annotation of them were conducted using Metascape website. GO analysis indicated that HNRNPC mainly plays an important role in the regulation of mRNA metabolic process, spliceosomal complex, and RNA binding (Figures 5(c) and 5(d)). Besides, the representative enriched KEGG pathways were spliceosome and oxidative phosphorylation pathways (Figures 5(e) and 5(f)). Moreover, GSEA was employed, and the top five significant associated pathways were G2M checkpoints, E2F targets, MYC targets, epithelial-mesenchymal transition (EMT), and mitotic spindle (Figure S3).

### 3.6. HNRNPC Is Intimately Correlated with Immune Cell Infiltration in Tumor Microenvironment of pRCC

**Figure 6:**
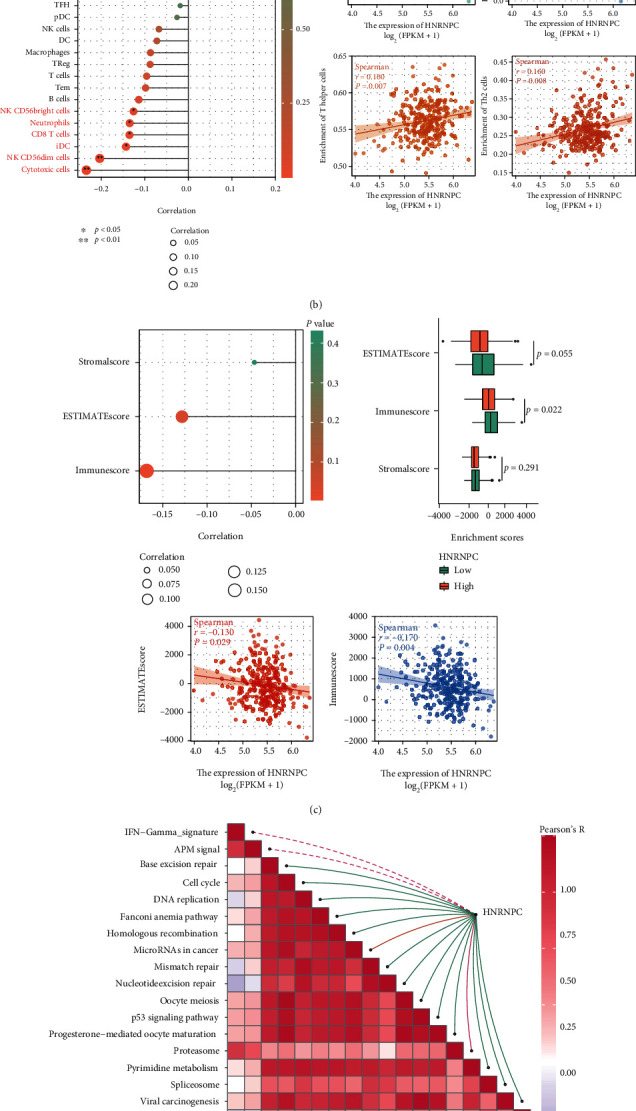
Relationship between HNRNPC mRNA expression and immune infiltration in the tumor microenvironment. (a) Heatmap of the correlation between HNRNPC expression and various immune cells and ESTIMATE score. (b) The infiltration level of cytotoxic cells, NK cells, immature dendritic cells (iDC), and CD8+ T cells. (c) The correlation and expression level between HNRNPC and ESTIMATE score. (d) HNRNPC was strongly correlated with the enrichment scores of immunotherapy-related signatures. (e) The correlation between HNRNPC and immunomodulators (PDCD1, CD274, LAG3, CTLA-4, TIGIT, HAVCR2, PDCD1LG2, and SIGLEC15) ^∗^*p* < 0.05; ^∗∗^*p* < 0.01; ^∗∗∗^*p* < 0.001; ns: no significant.

The complexity of tumor immune microenvironment (TIME) was an essential component of cancer development and dissemination [37, 38]. Therefore, ESTIMATE score of pRCC samples was calculated to uncover the relationship between HNRNPC and immune cell infiltration using ssGSEA methods, as illustrated in the heatmap (Figure 6(a)). The results showed that HNRNPC expression was negatively correlated with most immune cells' infiltration levels. Among them, HNRNPC obtained the strongest negative correlation with cytotoxic cells, NK cells, immature dendritic cells (iDC), and CD8+ T cells, which indicated itself a positive regulator of immunosuppression. Nevertheless, Spearman's analysis showed it was positively correlated with T helper (Th) cells and T helper 2 (Th2) cells (Figure 6(b)). Next, significantly lower and negative correlation with immune score were observed in the high-HNRNPC group compared to the low-HNRNPC group using the ESTIMATE method [39] (*p* = 0.022, Figure 6(c)). Notably, we also analyzed the correlations between HNRNPC level and a set of gene signatures which predict response to Immune Checkpoint Blocker (ICB) therapy, and the results suggested that HNRNPC was strongly correlated with the enrichment scores of immunotherapy-related signatures [25] (Figure 6(d)). Finally, the correlation between HNRNPC and immunomodulators (PDCD1, CD274, LAG3, CTLA-4, TIGIT, HAVCR2, PDCD1LG2, and SIGLEC15) is illustrated in Figure 6(e). To further evaluate the influence of HNRNPC on immune systems, the link between HNRNPC and several typical immune cell markers was analyzed in TIMER database (Table 3). The HNRNPC level turned out to be significantly associated with 15 immune cell markers out of 44. These findings partially support the negative relationship between HNRNPC expression and immune cell infiltration in pRCC.

### 3.7. Single-Cell Analysis Revealed the Distribution of HNRNPC in pRCC Tissues

**Figure 7:**
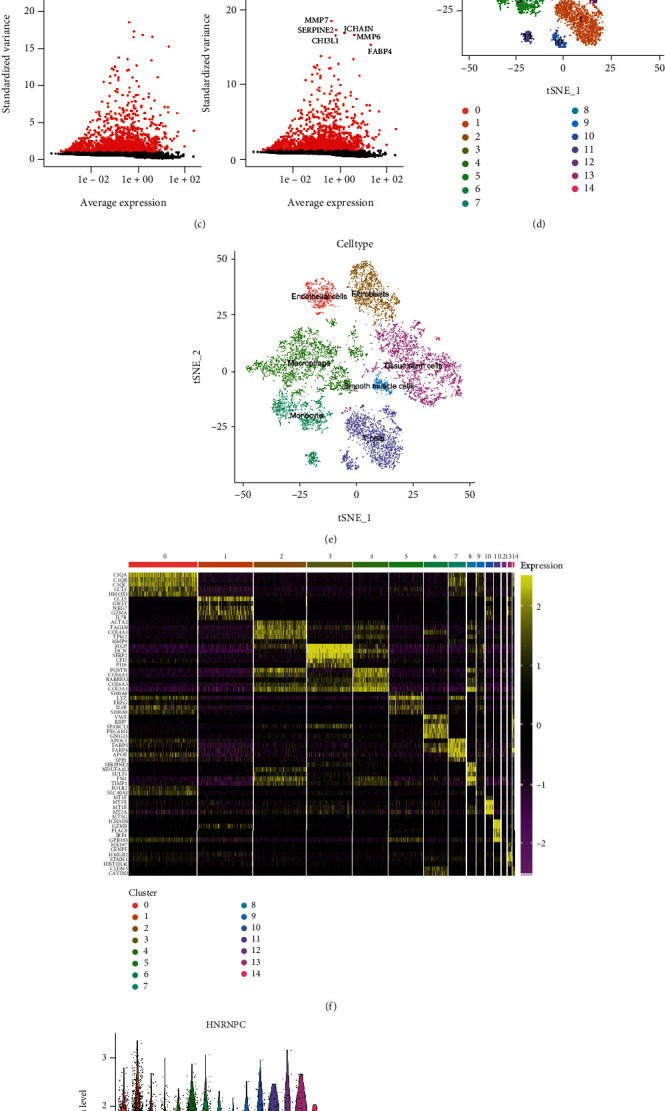
Single-cell analysis revealed the distribution of HNRNPC in pRCC tissues. (a) Quality control of pRCC single-cell RNA-seq samples. The number of gene expressions in each cell, the sum of gene expression, and the percentage of mitochondrial genes were illustrated. (b) The number of genes in the cells was positively correlated with the sum of gene expression, with a correlation of 0.93. (c) 3000 hypervariable genes from all the genes shown in red and the top 10 hypervariable genes. (D-E). The results of the cell cluster obtained by cell marker gene annotation were consistent with those obtained by “SingleR” package annotation. (f) Heatmap illustrated PCA unsupervised clustering in each compartment. (g, h) Distribution of HNRNPC in pRCC tissues obtained by tSNE algorithm.

In addition to bulk-RNA sequencing, we analyzed the immune microenvironment of pRCC with single-cell RNA-sequencing dataset GSE152938 [28]. Firstly, we performed quality control to ensure the reliability of cells for following analysis (Figure 7(a)). We found that the number of genes in cells was positively correlated with the count of gene expression (correlation coefficient = 0.8, Figure 7(b)). Then, we screened out 3000 highly variable genes and marked the top 10 genes in red (Figure 7(c)). Unsupervised clustering and principal component analysis (PCA) of each compartment gave rise to a total 15 clusters which were illustrated in a heatmap (Figures 7(d) and 7(f)). Later, we used “SingleR” package to annotate different cluster cells obtained by cell markers. As shown in Figure 7(e), pRCC samples could be mainly divided into endothelial cells, fibroblasts, macrophage, tissue stem cells, smooth muscle cells, monocyte, and T cells. Finally, we tried to explore the exact distribution of HNRNPC in pRCC tissues. The graphs obtained by tSNE algorithm revealed that expression of HNRNPC was mostly concentrated in macrophages, followed by monocyte and endothelial cells, which were consistent with the result of immune infiltration analysis (Figures 7(g) and 7(h)).

### 3.8. HNRNPC Played a Remarkable Oncogenic Role in pRCC

**Figure 8:**
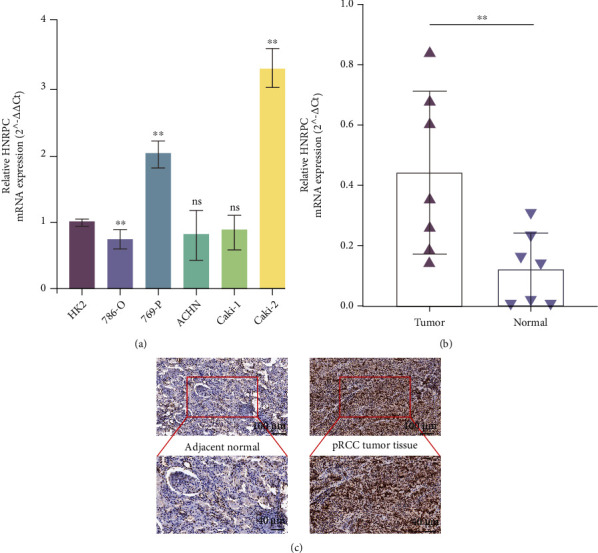
Confirmation of upregulated HNRNPC expression in pRCC. (a) qRT-PCR verified the expression level of HNRNPC in RCC cell lines. (b) qRT-PCR verified the expression level of HNRNPC in seven paired pRCC clinical samples. (c) IHC assays confirmed the tissue abundance in pRCC samples and normal renal samples.

**Figure 9:**
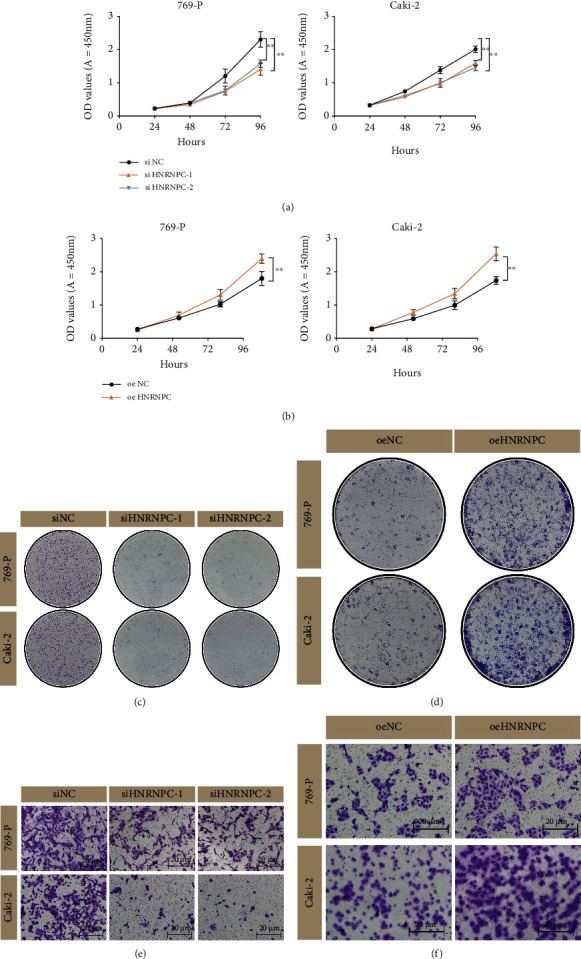
Experiment validation among the oncogenic role of HNRNPC in pRCC. (a, b). HNRNPC knockdown significantly weakened the proliferative capability of 769-P and Caki-2 cells, while overexpressing HNRNPC reversed the effect. (c, d) Colony formation assays of 769-P and Caki-2 cells. (e, f) HNRNPC decreased the metastatic ability of pRCC cells, while HNRNPC overexpression granted tumor cells much more aggressiveness. Scale bar: 20 *μ*m. ^∗∗^*p* < 0.05; ns: no significant.

To validate the encouraging findings achieved through bioinformatics analyses, in vitro experiments were conducted subsequently. First, mRNA expression of HNRNPC was detected in RCC cell lines and exhibited an obvious upregulated expression level in 769-P and Caki-2 (Figure 8(a)). In seven pRCC samples obtained from our cohort, HNRNPC mRNA level was statistically upregulated in tumor tissues than normal tissues (Figure 8(b)). Meanwhile, its tissue abundance was measured using IHC, which achieved consistent results (Figure 8(c)). Then, siRNA (siHNRNPC-1/siHNRNPC-2) and lentivirus (oeNC/oeHNRNPC) were utilized to accomplish HNRNPC knockdown and overexpression in pRCC cell lines, respectively. After transfecting siRNAs or lentivirus into 769-P and Caki-2 cells, HNRNPC expression levels were measured by qRT-PCR accordingly (Figure S4). The results of CCK-8 and colony formation assay indicated that HNRNPC knockdown significantly weakened the proliferative capability of both cell lines, especially Caki-2 cells, while overexpressing HNRNPC reversed the effect (Figures 9(a)–9(d)). Furthermore, transwell migration assay demonstrated that silencing HNRNPC decreased the metastatic ability of pRCC cells, while HNRNPC overexpression granted tumor cells much more aggressiveness (Figures 9(e) and 9(f)). Taken together, these results showed that elevated HNRNPC could promote the proliferation and invasiveness of pRCC cells.

## 4. Discussion

Papillary renal cell carcinoma accounts for 7%-14% of renal cell carcinomas and is not uncommon to see in clinical patients [7]. Limited by its relatively small number of cases, pRCC has not been thoroughly investigated in both molecular mechanism research and large-scale clinical trials. m^6^A RNA methylation, a typical biological process that plays an indispensable role in posttranscriptionally regulating splicing, stability, transportation, and translation of targeted mRNAs, has been shown to participate in the modulation of numerous tumors [12, 40]. For this reason, much attention has been paid to the regulation of m^6^A RNA methylation itself [41–43]. Especially, Zaccara et al. reviewed the key function of epitranscriptome in affecting gene expression and made a comprehensive summary to three main methods of regulating m^6^A effects on mRNA—reading, writing, and erasing [44]. By means of bioinformatics analyses, Yang et al. and Chen et al. identified significant prognostic biomarkers and risk gene signature in pRCC to emphasize the pivotal role of m^6^A RNA methylation regulators [45, 46]. Similarly, Sun and colleagues constructed a prognostic risk signature with three m^6^A RNA methylation regulators and found these genes could precisely predict the survival outcomes of pRCC patients [47]. Unfortunately, despite the growing number of database-dependent research on m^6^A RNA methylation regulation, there is still a lack of advanced and convictive studies to interpret its role in the origination and progression of pRCC.

In the present study, through comprehensive bioinformatics analyses and reliable fundamental experiments, HNRNPC was identified as a significant m^6^A mRNA methylation regulator to promote malignant phenotypes of pRCC and lower the survival expectation of pRCC patients. HNRNPC, known as heterogeneous nuclear ribonucleoprotein C, belongs to the subfamily of ubiquitously expressed heterogeneous nuclear ribonucleoproteins (hnRNPs), which are binding proteins to heterogeneous nuclear RNA (hnRNA). These proteins are associated with mRNA precursors in the nucleus and have been verified to influence the pre-mRNA processing, structure switching, and other aspects of mRNA metabolism. Detailly, multiple functions for hnRNPs in the nucleus and cytoplasm include mRNA splicing, stability, turnover, export, and translation, which grant themselves frequent involvement of various mRNA regulatory events [48]. Over years, the regulatory nature of hnRNPs has exerted unignorable impacts on the progression of diverse diseases, especially malignant tumors. By revealing that hnRNPs control the alternative splicing of pyruvate kinase mRNA in tumorigenesis, Chen et al. offered a novel sight into the molecular mechanism of how hnRNPs regulate cancer proliferation from a metabolic perspective [49]. Kedzierska et al. summarized that hnRNPs regulate all levels of expression in most apoptotic genes, which demonstrates their role of apoptosis regulator in cancers and significance in anticancer therapy [50]. These studies shed light on the crucial regulatory capability of hnRNPs on the tumor progression and cancer development.

In the beginning, our study illustrated the tight association between HNRNPC expression and clinical outcome in pRCC. Analyses on adequate samples from several databases collectively showed high expression of HNRNPC in pRCC tumoral samples. Logistic regression analysis then further revealed that higher level HNRNPC was linked to poorer survival and more malignant phenotype in pRCC patients. Notably, pan-cancer analysis showed that HNRNPC was overexpressed in tumoral tissues compared to normal tissues in most common cancers and was also strongly correlated with poor clinical and pathological features. Numerous studies have confirmed this finding [51–53]. Inspired by the firm relationship between HNRNPC level and clinical outcomes, we next established the prognostic role of HNRNPC as a dependable biomarker in pRCC patients. The nomogram and calibration curves of prediction model demonstrated that HNRNPC expression was well qualified to predict tumor stage and overall survival of pRCC patients as an independent risk factor. Similar findings were also observed in other studies. Wang et al. found that the expression of HNRNPC was significantly increased in prostate cancer tissues and positively correlated with the T stage, N stage, Gleason score, and PSA level [52]. Guo and colleagues also showed that HNRNPC was highly expressed in lung adenocarcinoma (LUAD) tissues and significantly related to smoking history, lymph node metastasis, and poor prognosis. Therefore, HNRNPC expression was an independent prognostic factor for LUAD patients [53]. Reversely, Wang et al. found that in glioblastoma multiforme (GBM), higher expression of HNRNPC was associated with a better prognosis [54]. This may be explained by the heterogeneity of tumors and diverse operating ways of hnRNPs in various cancers [14].

Since HNRNPC was reported to regulate cancer progression through diverse systems [21, 55], the specific mechanisms of its tumor-promoting role in pRCC came as an unavoidable concern. For the reason, the subsequent GSVA, functional enrichment, and pathway annotation were conducted and meant to uncover the underlying driving event controlled by HNRNPC in pRCC development. As expected, results from GO and KEGG analyses turned out to be tightly associated with the pre-mRNA processing and mRNA metabolism, and HNRNPC fundamentally regulated pRCC by serving as a vital composition of spliceosome. Moreover, GSVA revealed significantly differentially expressed pathways in paired pRCC samples, and those with Hallmarks of “MYC/E2F targets,” “G2M checkpoints,” and “DNA repair” stood out as highly expressed ones in tumor tissues. Published studies have confirmed that c-Myc mRNA may serve as a potential target of hnRNPs. Huang et al. reported that in the Linc-RoR-mediated c-Myc expression system, HNRNPI could be the basic equipment which was essential for the interactions between them [56]. Interestingly, David and colleagues found that c-Myc transcriptionally upregulated the protein level of several hnRNPs, thus dysregulating their alternative splicing of M2 subtype of pyruvate kinase (PKM2) mRNA to influence tumor cell proliferation [57]. These findings indicated a potential mutually regulatory relationship between c-Myc and hnRNPs and emphasized their important and inseparable role in tumor development. G2/M cell cycle checkpoints, also known as DNA damage checkpoints, serve to prevent the cell from entering mitosis (M-phase) with genomic DNA damage. In this biological process, p53 is commonly activated by DNA damage checkpoint kinases to regulate the G1/S and G2/M checkpoints simultaneously [58]. By turning attention to the RNA binding proteins, Cannell et al. revealed that hnRNPA0 protein was a major substrate of the checkpoint kinase MK2, and it enforced cell cycle arrest and promoted resistance to DNA damaging chemotherapy through targeting mRNA which controlled the G2/M DNA damage checkpoints [59]. Similarly, hnRNPA0 was reported by Konishi et al. to inhibit the apoptosis and promote mitosis through the maintenance of G2/M-phase, thus playing a tumor promotive role in colorectal cancer cells [60]. To our knowledge, this study was the first to bring focus on the relationship between HNRNPC and G2/M checkpoint pathway in tumor-related research. We hoped to offer a novel insight into uniting the m^6^A RNA methylation with cell cycle control as a promising research topic in the future.

According to previous studies, HNRNPC has been reported to regulate immunosuppressive events in tumor microenvironment [61, 62], and we conducted comparable analyses on HNRNPC to uncover its role in immune systems. Among them, representative methods including ESTIMATE algorithm and ssGSEA were applied to evaluate the immune infiltration level. In accordance with previous studies, HNRNPC was proved to be a significant suppressor of internal immune activity in pRCC [46]. To summarize, we assumed that HNRNPC expression did favor to the prediction of the immune infiltrating levels of TME in pRCC and acquired applicable clinical value.

As m^6^A RNA methylation gains increasing popularity in renal cancer research, a great deal of functional genes and pathways was considered to participate in this process in pRCC. Through comprehensive bioinformatics analyses, Yang et al. [45], Chen et al. [46], and Sun et al. [47] separately constructed the m^6^A RNA methylation gene-based risk signature as prognostic biomarker for pRCC. Interestingly, HNRNPC appeared to be the mostly discussed hub gene in these congeneric studies. In these studies, it showed solid clinical correlation and independent prognostic capability, which was consistent with our findings to a great extent. Despite the emerging trend of online research, there is still an urgent call for more convincing investigation. To make a complementary study, we additionally performed fundamental experiments through molecular and cellular biology methods. qPCR and IHC analysis distinctly verified the predictive expression pattern of HNRNPC in pRCC. HNRNPC mRNA level and protein level were statistically upregulated in tumor tissues than normal tissues. Afterwards, the results of functional assays proved the pronounced oncogenic role of HNRNPC in pRCC cell lines. Taken together, these results robustly established the tumor-associated role of HNRNPC in pRCC.

## 5. Conclusion

Our study conducted comprehensive bioinformatics analyses on m^6^A mRNA methylation regulators, and HNRNPC turned out to be an indispensable risk factor of pRCC. As a reader, HNRNPC exerts profound impact on tumor progression and clinical outcomes in pRCC patients, and it could be a potential target for more promising treatment in the future.

## Figures and Tables

**Table 1:** Correlation between HNRNPC expression and clinicopathological characteristics in pRCC.

Characteristic	Low expression of HNRNPC	High expression of HNRNPC	*p* value
*n*	144	145	
Age, *n* (%)			0.189
≤60	60 (21%)	73 (25.5%)	
>60	82 (28.7%)	71 (24.8%)	
Gender, *n* (%)			0.763
Female	40 (13.8%)	37 (12.8%)	
Male	104 (36%)	108 (37.4%)	
T stage, *n* (%)			0.043
T1	75 (37.3%)	64 (31.8%)	
T2	15 (7.5%)	11 (5.5%)	
T3	11 (5.5%)	24 (11.9%)	
T4	0 (0%)	1 (0.5%)	
N stage, *n* (%)			<0.001
N0	70 (45.8%)	62 (40.5%)	
N1	2 (1.3%)	17 (11.1%)	
N2	1 (0.7%)	1 (0.7%)	
M stage, *n* (%)			0.171
M0	101 (48.3%)	99 (47.4%)	
M1	2 (1%)	7 (3.3%)	
Stage, *n* (%)			0.010
Stage I	76 (38.4%)	62 (31.3%)	
Stage II	12 (6.1%)	9 (4.5%)	
Stage III	8 (4%)	21 (10.6%)	
Stage IV	2 (1%)	8 (4%)	
Age, mean ± SD	62.72 ± 10.6	60.44 ± 12.99	0.106

**Table 2:** Logistic regression analysis between HNRNPC expression and clinicopathological characteristics.

Characteristics	Total (*N*)	Odds ratio (OR)	*p* value
Age (>60 vs. ≤60)	286	0.712 (0.445-1.133)	0.153
Gender (female vs. male)	289	0.891 (0.527-1.501)	0.664
T stage (T3 & T4 vs. T1 & T2)	287	1.904 (1.073-3.439)	0.030
N stage (N1 & N2 vs. N0)	77	4.416 (1.533-14.858)	0.009
M stage (M1 vs. M0)	209	3.571 (0.839-24.361)	0.118

**Table 3:** Correlations between HNRNPC and gene markers of immune cells in TIMER database.

Immune cell type	Gene marker	TCGA-KIRP cohort			
		None		Purity	
		Cor	*p*	Cor	*p*
B cell	CD19	0.082	ns	0.073	ns
	CD38	0.148	^∗∗^	0.164	^∗∗^

CD8+ T cell	CD8A	-0.008	ns	0	ns
	CD8B	-0.082	ns	-0.077	ns

Tfh	CXCR5	0.029	ns	0.043	ns
	ICOS	0.038	ns	0.036	ns
	BCL-6	0.336	^∗∗^	0.356	^∗∗^

Th1	IL12RB2	0.074	ns	0.048	ns
	IL27RA	0.36	^∗∗^	0.122	^∗∗^

Th2	CCR3	0.077	ns	0.091	ns
	STAT6	0.275	^∗∗^	0.3	^∗∗^
	GATA-3	0.011	ns	0.026	ns

Th9	TGFBR2	0.434	^∗∗^	0.466	^∗∗^
	IRF4	0.046	ns	0.062	ns
	PU.1 (SPI1)	-0.101	ns	-0.106	ns

Th17	IL-21R	0.061	ns	0.065	ns
	IL-23R	0.252	^∗∗^	0.229	^∗∗^
	STAT3	0.487	^∗∗^	0.509	^∗∗^

Th22	CCR10	0.027	ns	0.022	ns
	AHR	0.345	^∗∗^	0.371	^∗∗^

Treg	FOXP3	0.139	^∗∗^	0.14	^∗∗^
	CCR8	0.095	ns	0.101	ns
	IL2RA	0.226	^∗∗^	0.262	^∗∗^

T cell exhaustion	PD-1	-0.039	ns	-0.057	ns
	CTLA4	-0.035	ns	-0.049	ns

Macrophage	CD68	0.034	ns	0.066	ns
	CD11b (ITGAM)	0.042	ns	0.034	ns

M1	CD83	0.048	ns	0.057	ns
	NOS2	0.114	ns	0.114	ns
	ROS1	0.09	ns	0.096	ns

M2	ARG1	0.127	^∗∗^	0.164	^∗∗^
	MRC1	0.153	^∗∗^	0.168	^∗∗^

TAM	HLA-G	-0.03	ns	0.004	ns
	CD80	0.107	ns	0.117	ns
	CD86	0.048	ns	0.06	ns

Monocyte	CD14	-0.03	ns	-0.021	ns
	CD16 (FCGR3A)	0.074	ns	0.065	ns

NK	XCL1	-0.039	ns	-0.02	ns
	KIR3DL1	-0.048	ns	-0.047	ns
	CD7	-0.105	ns	-0.09	ns

Neutrophil	CD15 (FUT4)	0.325	^∗∗^	0.356	^∗∗^
	MPO	-0.081	ns	-0.055	ns

DC	CD1C	0.148	^∗∗^	0.178	^∗∗^
	CD141 (THBD)	0.326	^∗∗^	0.342	^∗∗^

Tfh: follicular helper T cell; Th: T helper cell; Treg: regulatory T cell; TAM: tumor-associated macrophage; NK: natural killer cell; DC: dendritic cell; none: correlation without adjustment; purity: correlation adjusted for tumor purity; Cor: R value of Spearman's correlation; ns: no significant; ^∗∗^*p* < 0.05.

## Data Availability

The data we used to support the findings of the study are mentioned in this study. Other relevant data that support the findings of this study are available from the corresponding author upon reasonable request.

## Supplementary Materials

Supplementary Materials Figure S1: genetic mutation frequency of HNRNPC. (A, B) cBioPortal database was investigated to obtain the mutation frequencies and status of genetic alterations of HNRNPC across multiple cancer types. (C) The mutation frequency sites and case numbers of the HNRNPC genetic alterations. (D) Correlation between copy number variation (CNV) of HNRNPC and the infiltration levels of immune cells. Figure S2: construction of PPI network. (A) GeneMANIA databases were used to establish the PPI network. (B) Construction of PPI network with Metascape website and illustrated by clusters and *p* value. Figure S3: GSEA of HNRNPC-related genes in TCGA cohort. (A) Heatmap of top 50 coexpressed genes with HNRNPC. (B) GSEA showed top five significant pathways associated. Figure S4: qRT-PCR validation of the relative expression of HNRNPC. (A) The relative expression of HNRNPC in 769-P cells transfected with si-NC or si-HNRNPC. (B) The relative expression of HNRNPC in Caki-2 cells transfected with si-NC or si-HNRNPC. (C) The relative expression of HNRNPC in 769-P cells transfected with negative control lentivirus or overexpression lentivirus. (D) The relative expression of HNRNPC in Caki-2 cells transfected with negative control lentivirus or overexpression lentivirus. The data are presented as the mean ± SD; ^∗∗^*p* < 0.05. Table S1: oligonucleotide sequences used in this research. Table S2: univariate and multivariate Cox regression analyses of HNRNPC in overall survival (OS).
