# Supplementary material for: Essential m6A Methylation Regulator HNRNPC Serves as a Targetable Biomarker for Papillary Renal Cell Carcinoma
Source: J Oncol. 2022 Apr 23;2022:9411692. doi: 10.1155/2022/9411692 (PMC9056237; doi:10.1155/2022/9411692)
Supplement: Supplementary Materials — Figure S1: genetic mutation frequency of HNRNPC. (A, B) cBioPortal database was investigated to obtain the mutation frequencies and status of genetic alterations of HNRNPC across multiple cancer types. (C) The mutation frequency sites and case numbers of the HNRNPC genetic alterations. (D) Correlation between copy number variation (CNV) of HNRNPC and the infiltration levels of immune cells. Figure S2: construction of PPI network. (A) GeneMANIA databases were used to establish the PPI network. (B) Construction of PPI network with Metascape website and illustrated by clusters and p value. Figure S3: GSEA of HNRNPC-related genes in TCGA cohort. (A) Heatmap of top 50 coexpressed genes with HNRNPC. (B) GSEA showed top five significant pathways associated. Figure S4: qRT-PCR validation of the relative expression of HNRNPC. (A) The relative expression of HNRNPC in 769-P cells transfected with si-NC or si-HNRNPC. (B) The relative expression of HNRNPC in Caki-2 cells transfected with si-NC or si-HNRNPC. (C) The relative expression of HNRNPC in 769-P cells transfected with negative control lentivirus or overexpression lentivirus. (D) The relative expression of HNRNPC in Caki-2 cells transfected with negative control lentivirus or overexpression lentivirus. The data are presented as the mean ± SD; ∗∗p < 0.05. Table S1: oligonucleotide sequences used in this research. Table S2: univariate and multivariate Cox regression analyses of HNRNPC in overall survival (OS). [file 9411692.f1.docx]

Figure S1: Genetic mutation frequency of HNRNPC.

(A-B). cBioPortal database was investigated to obtain the mutation frequencies and status of genetic alterations of HNRNPC across multiple cancer types. (C). The mutation frequencies sites and case numbers of the HNRNPC genetic alterations. (D). Correlation between copy number variation (CNV) of HNRNPC and the infiltration levels of immune cells.


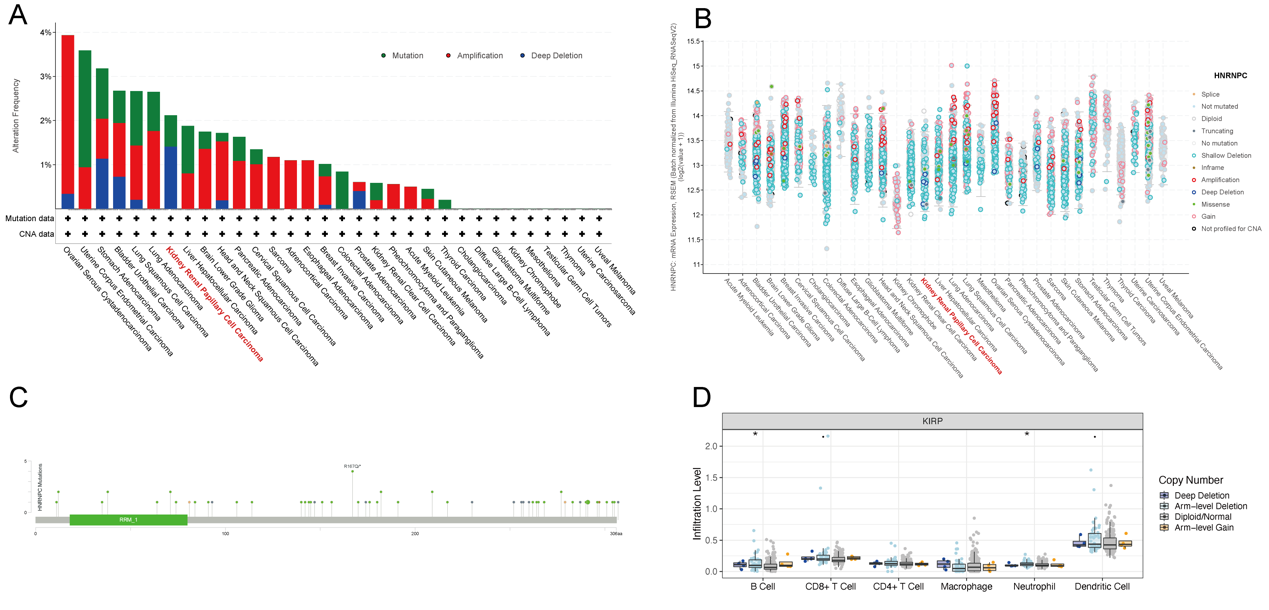


Figure S2: Construction of PPI network.

(A). GeneMANIA databases were used to establish the PPI network. (B). Construction of PPI network with Metascape website and illustrated by clusters and p-value.


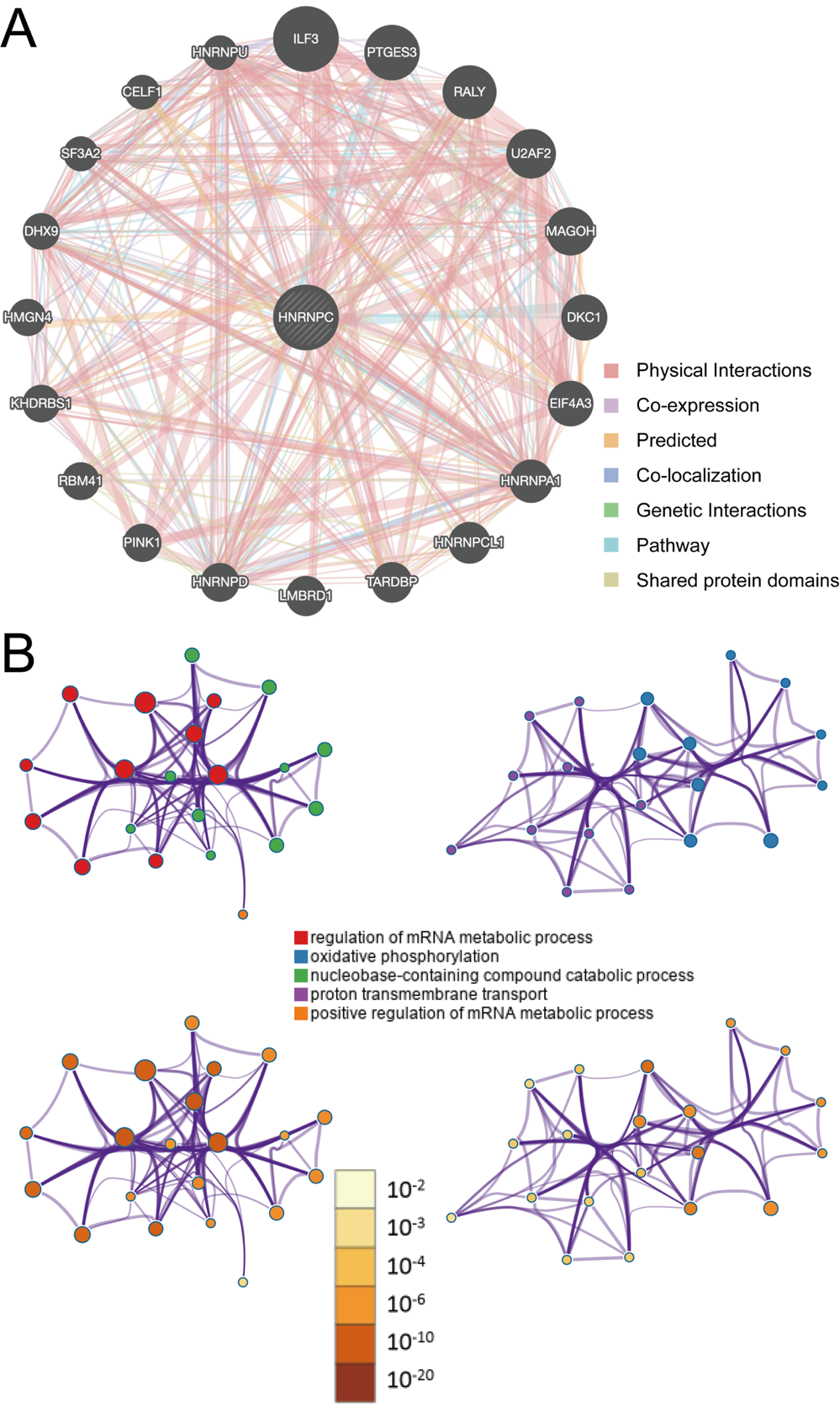


Figure S3: GSEA analysis of HNRNPC-related genes in TCGA cohort.

(A). Heatmap of top 50 co-expressed genes with HNRNPC. (B). GSEA analysis showed top five significant pathways associated.


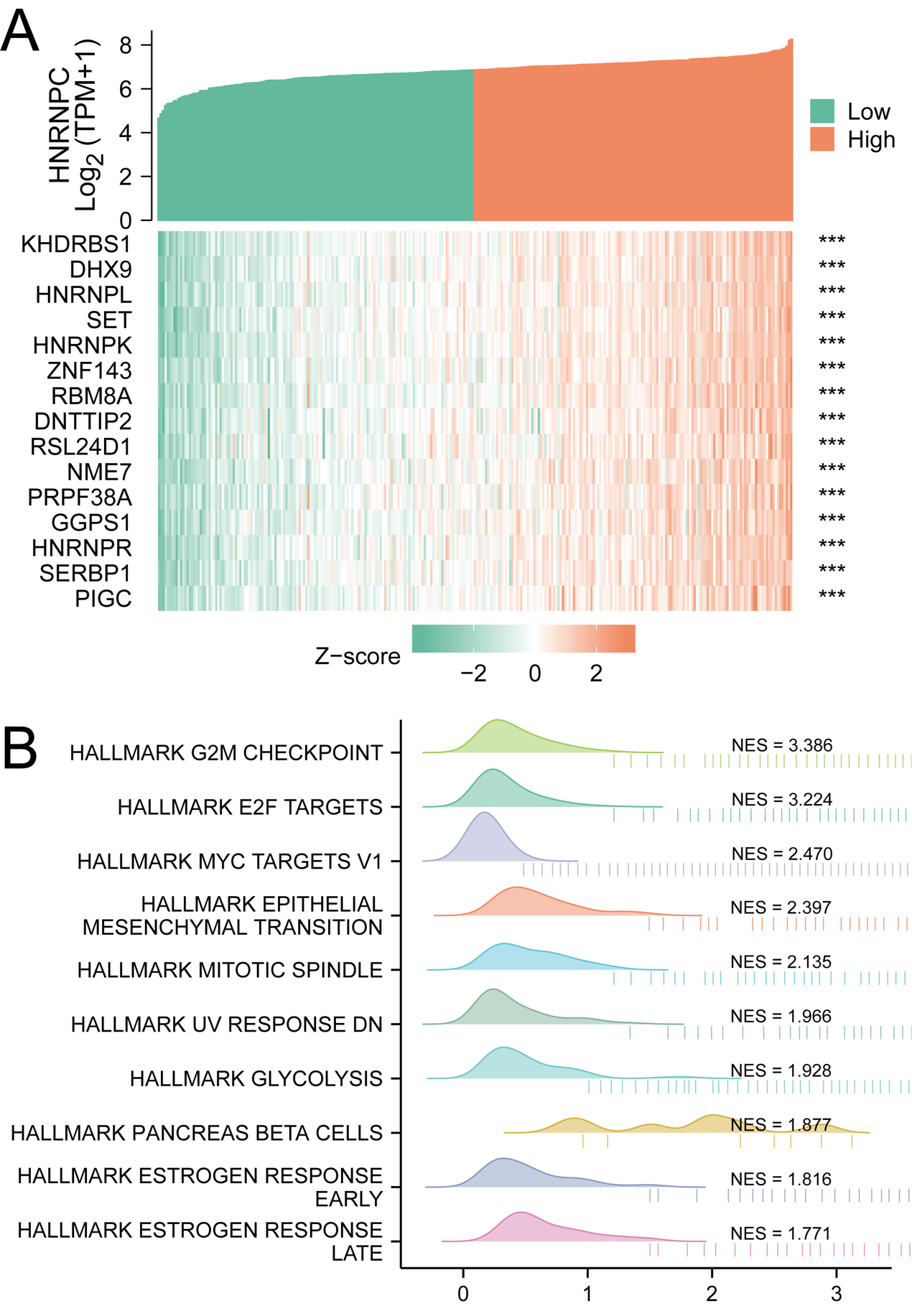


Figure S4: qRT-PCR validation of the relative expression of HNRNPC.

(A). The relative expression of HNRNPC in 769-P cells transfected with si-NC or si-HNRNPC. (B). The relative expression of HNRNPC in Caki-2 cells transfected with si-NC or si-HNRNPC. (C). The relative expression of HNRNPC in 769-P cells transfected with negative control lentivirus or overexpression lentivirus. (D). The relative expression of HNRNPC in Caki-2 cells transfected with negative control lentivirus or overexpression lentivirus.

The data are presented as the mean ± SD; **: p<0.05.


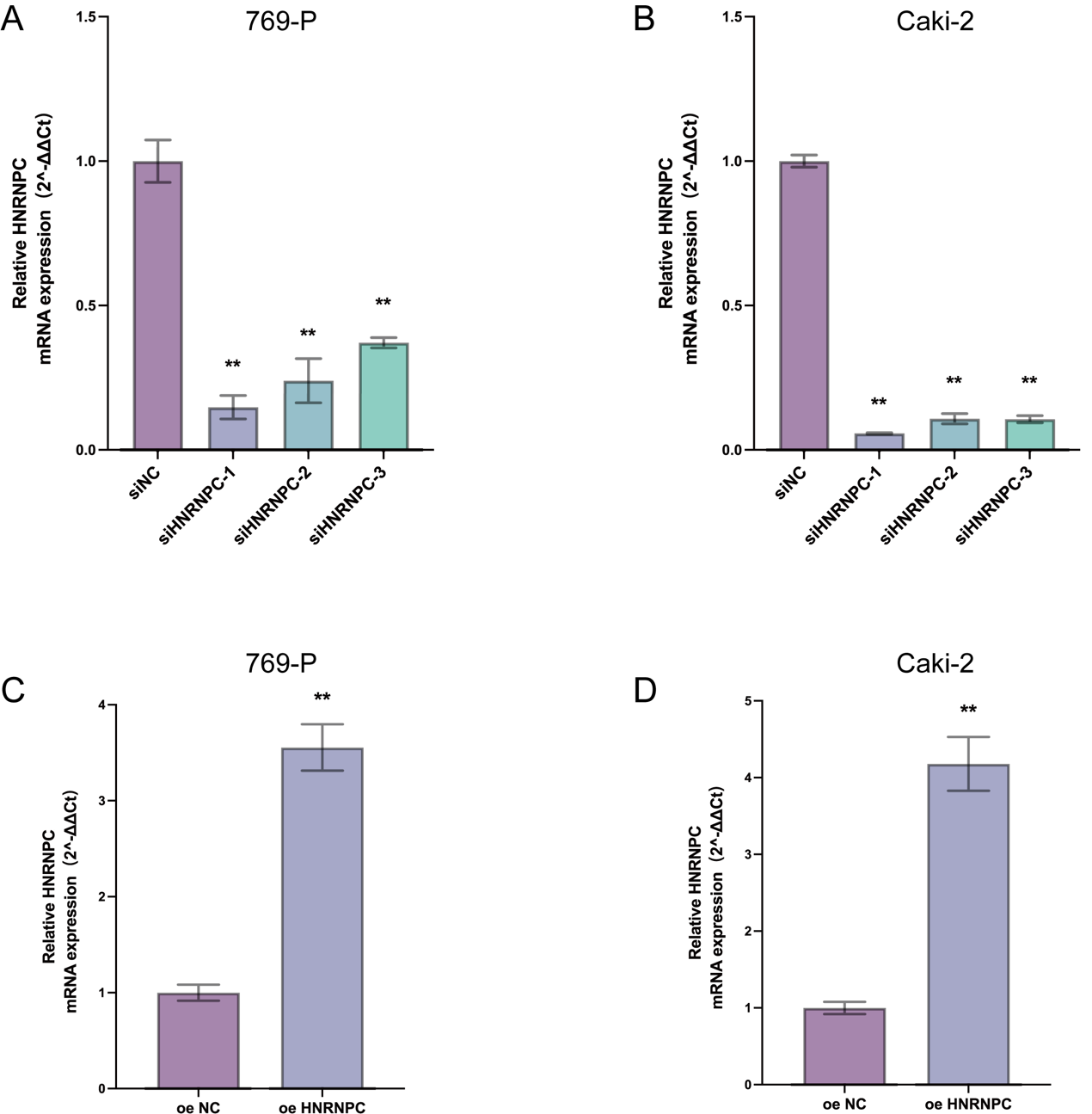


**Table S1 Oligonucleotide sequences used in this research**

| Primers | Sequences | |
| --- | --- | --- |
| HNRNPC | Forward | GGAGATGTACGGGTCAGTAACA |
|  | Reverse | CCCGAGCAATAGGAGGAGGA |
| β-actin | Forward | ATGACTTAGTTGCGTTACACC |
|  | Reverse | GACTTCCTGTAACAACGCATC |
| siRNA-NC | Forward | UUCUCCGAACGUGUCACGUTT |
|  | Reverse | ACGUGACACGUUCGGAGAATT |
| siRNA-HNRNPC-1 | Forward | GCAGGUGUGAAACGAUCUGTT |
|  | Reverse | CAGAUCGUUUCACACCUGCTT |
| siRNA-HNRNPC-2 | Forward | GCAGUAGAGAUGAAGAAUGTT |
|  | Reverse | CAUUCUUCAUCUCUACUGCTT |
| siRNA-HNRNPC-3 | Forward | UGAAGAAAGAUGAGACUAATT |
|  | Reverse | UUAGUCUCAUCUUUCUUCATT |

**Table S2 Univariate and multivariate Cox regression analysis of HNRNPC in overall survival (OS)**

| Characteristics | Total(N) | Univariate analysis | |  | Multivariate analysis | |
| --- | --- | --- | --- | --- | --- | --- |
|  |  | Hazard ratio (95% CI) | P value |  | Hazard ratio (95% CI) | P value |
| Pathologic T stage | 286 |  |  |  |  |  |
| T1 | 192 | Reference |  |  |  |  |
| T2 | 33 | 3.011 (1.244-7.285) | **0.014** |  | 0.455 (0.036-5.759) | 0.543 |
| T3&T4 | 61 | 6.846 (3.423-13.690) | **<0.001** |  | 0.348 (0.021-5.786) | 0.462 |
| Pathologic N stage | 77 |  |  |  |  |  |
| N0 | 49 | Reference |  |  |  |  |
| N1&N2 | 28 | 5.003 (2.062-12.140) | **<0.001** |  | 0.857 (0.148-4.967) | 0.863 |
| Pathologic M stage | 104 |  |  |  |  |  |
| M0 | 95 | Reference |  |  |  |  |
| M1 | 9 | 114.966 (22.481-587.925) | **<0.001** |  | 36.764 (3.719-363.429) | **0.002** |
| Clinical stage | 198 |  |  |  |  |  |
| Stage I&II | 159 | Reference |  |  |  |  |
| Stage III&IV | 39 | 9.489 (4.596-19.592) | **<0.001** |  | 5.656 (0.398-80.326) | 0.201 |
| HNRNPC | 288 |  |  |  |  |  |
| Low | 144 | Reference |  |  |  |  |
| High | 144 | 2.204 (1.149-4.228) | **0.017** |  | 1.142 (0.312-4.178) | 0.841 |
